# Items

**Published:** 1883-01

**Authors:** 


### Items.

### EDITORIAL NOTE.

It will be seen that, from the cover of this first number of THE CHICAGO MEDICAL JOURNAL AND EXAMINER for 1883, the names of its editors have been omitted. The change is one which has long been contemplated, and which will, it is hoped, be found useful and practicable in the further conduct of this organ of the Press Association. Hereafter its management will be impersonal, and THE JOURNAL will rest upon its intrinsic merits for a continuation of that favor on the part of the profession which has placed it in its present position. That position is, as regards Western journalism, one of unprecedented financial and general success.

DR. W. T. BELFIELD, whose interesting letters from Germany have from time to time appeared in the JOURNAL AND EXAMINER, will deliver the Cartwright course of lectures of the Alumni Association of the College of Physicians and Surgeons of New York. The lectures will be given on the evenings of February 19, 21, 23 and 26, in the Y. M. C. A. hall, opposite the college. The subjects will be the relations of micro-organisms to disease.

RUSH MEDICAL COLLEGE has honored itself, and conferred a deserved compliment upon Dr. E. Fletcher Ingals by electing him Professor of Laryngology.

IN consequence of a delay in preparing its illustrations the supplementary paper by Dr. H. D, Schmidt, of New Orleans, on the Bacillus Tuberculosis is deferred to the next issue of the JOURNAL. The matter is already in type and will certainly appear in the February number.

